# Restraining *Staphylococcus aureus* Virulence Factors and *Quorum Sensing* through Lactic Acid Bacteria Supernatant Extracts

**DOI:** 10.3390/antibiotics13040297

**Published:** 2024-03-25

**Authors:** Myriam Anabel Díaz, Esteban Gabriel Vega-Hissi, María Amparo Blázquez, María Rosa Alberto, Mario Eduardo Arena

**Affiliations:** 1 Instituto Superior de Investigaciones Biológicas (INSIBIO, CONICET-UNT), Chacabuco 461, San Miguel de Tucumán CP 4000, Argentina; anabel.tucuman@gmail.com; 2 Instituto Multidisciplinario de Investigaciones Biológicas (IMIBIO-SL), Facultad de Química, Bioquímica y Farmacia, Universidad Nacional de San Luis, Ejército de Los Andes 950, San Luis CP 5700, Argentina; egvega@unsl.edu.ar; 3 Departament de Farmacologia, Facultat de Farmàcia i Ciències de l’Alimentació, Universitat de València, Avd. Vicent Andrés Estellés s/n, Burjasot, 46100 Valencia, Spain; 4 Facultad de Bioquímica, Química y Farmacia, Universidad Nacional de Tucumán (UNT), Ayacucho 471, San Miguel de Tucumán CP 4000, Argentina; 5 Instituto de Biotecnología Farmacéutica y Alimentaria (INBIOFAL, CONICET-UNT), Avenida Kirchner 1900, San Miguel de Tucumán CP 4000, Argentina

**Keywords:** biofilm, *quorum sensing*, lactic acid bacteria, chloroform extract, *Staphylococcus aureus*

## Abstract

The escalating prevalence of antibiotic-resistant bacteria poses a grave threat to human health, necessitating the exploration of novel alternatives to conventional antibiotics. This study investigated the impact of extracts derived from the supernatant of four lactic acid bacteria strains on factors contributing to the pathogenicity of three *Staphylococcus aureus* strains. The study evaluated the influence of lactic acid bacteria supernatant extracts on the growth, biofilm biomass formation, biofilm metabolic activity, and biofilm integrity of the *S. aureus* strains. Additionally, the impact on virulence factors (hemolysin and coagulase) was examined. Gas chromatography coupled with mass spectrometry was used to identify the bioactive compounds in the extracts, while molecular docking analyses explored potential interactions. Predominantly, the extracts contain eight 2,5-diketopiperazines, which are cyclic forms of peptides. The extracts demonstrated inhibitory effects on biofilm formation, the ability to disrupt mature biofilms, and reduce the biofilm cell metabolic activity of the *S. aureus* strains. Furthermore, they exhibited the ability to inhibit α-hemolysin production and reduce coagulase activity. An in silico docking analysis reveals promising interactions between 2,5-diketopiperazines and key proteins (SarA and AgrA) in *S. aureus*, confirming their antivirulence and antibiofilm activities. These findings suggest that 2,5-diketopiperazines could serve as a promising lead compound in the fight against antibiotic-resistant *S. aureus*.

## 1. Introduction

Bacteria with antimicrobial resistance are found in animals, the environment, and humans. They can be transmitted between people, from animals to people, or even through foods of animal origin. Special attention is paid to microorganisms included in the ESKAPE list, which include *Enterococcus faecium*, *Staphylococcus aureus*, *Klebsiella pneumoniae*, *Acinetobacter baumannii*, *Pseudomonas aeruginosa*, and *Enterobacter* spp., and the research is aimed at minimizing the morbidity and mortality from these infectious agents [1].

In recent years, methicillin-resistant *S. aureus* (MRSA) has gained relevance as an etiological agent due to its rapid spread and the number of deaths it causes per year. It is a bacterium that is commonly found on the skin. However, it becomes pathogenic in immunodeficient or immunosuppressed patients [2]; it is the most common cause of infections associated with indwelling medical devices mediated by biofilms [3] and is responsible for skin infections, endocarditis, pneumonia, and bacteremia [4].

On the other hand, *S. aureus* represents a complication for human health and animals, capable of causing mastitis in ruminants, causing the deterioration of the animal’s health with a consequent reduction in milk production, leading to economic losses [5]. Furthermore, this bacterium can produce a wide variety of virulence factors, including the production of toxins such as α-hemolysin, enzymes such as coagulase, and pigments such as staphyloxanthin, and the production of biofilms [6].

Biofilms are defined as a community of microorganisms embedded in a matrix of extracellular polymers and associated with a surface that can be biotic or abiotic [7]. It has been observed that to eradicate bacteria in biofilms, antibiotic concentrations 1000 times higher than those used for bacteria in suspension are needed [8]. Among the possible causes for this high resistance are the high cell density that allows a greater exchange of mobile genetic elements (such as antibiotic resistance genes); the slower bacterial growth, which leads to a lower metabolic rate; and the presence of the extracellular matrix, which makes the diffusion of antibiotics difficult [9].

A communication process, called *quorum sensing* (QS), regulates the production of these virulence factors by cells based on the production, secretion, and detection of autoinducing signals (AIs). When these molecules reach a concentration threshold, a signaling cascade regulates the genetic expression of virulence factors [10]. In the case of *S. aureus*, the central quorum regulatory system is the accessory gene regulatory system (*agr*) [11].

The search for molecules capable of interfering with this communication system and, therefore, with the virulence of this bacteria has been proposed as a potential alternative to antibiotic therapy. Unlike conventional antibacterials and disinfectants, instead of killing the bacteria, this strategy that uses QS inhibitors that can block intercellular communication and significant limit the expression of phenotypes, such as biofilm formation and virulence factor production, while reducing the likelihood of resistance development [12].

Several studies have shown that lactic acid bacteria could interfere with QS, biofilm formation, and pathogen survival. They can also interfere with the integrity/quality of biofilms, leading to pathogen eradication. Some of these molecular mechanisms include the secretion of antagonistic substances (e.g., surfactants, bacteriocins, exopolysaccharides, organic acids, lactic acid, and fatty acids) and the generation of environmental conditions unfavorable for pathogens (e.g., pH alterations and competition for surfaces and nutrients) [12,13].

This work aimed to evaluate the ability of low polarity compounds present in the chloroform extract obtained from the supernatant of lactic acid bacteria to inhibit the production of various virulence factors of *S. aureus* (including biofilms). In addition, the main compounds of these extracts were chemically identified, and molecular docking tests were carried out with two receptor proteins (SarA and AgrA) and regulators of the QS of this bacterium.

## 2. Results

### 2.1. Effect of Extracts on Bacterial Growth and Biofilm Formation

**Figure 1:**
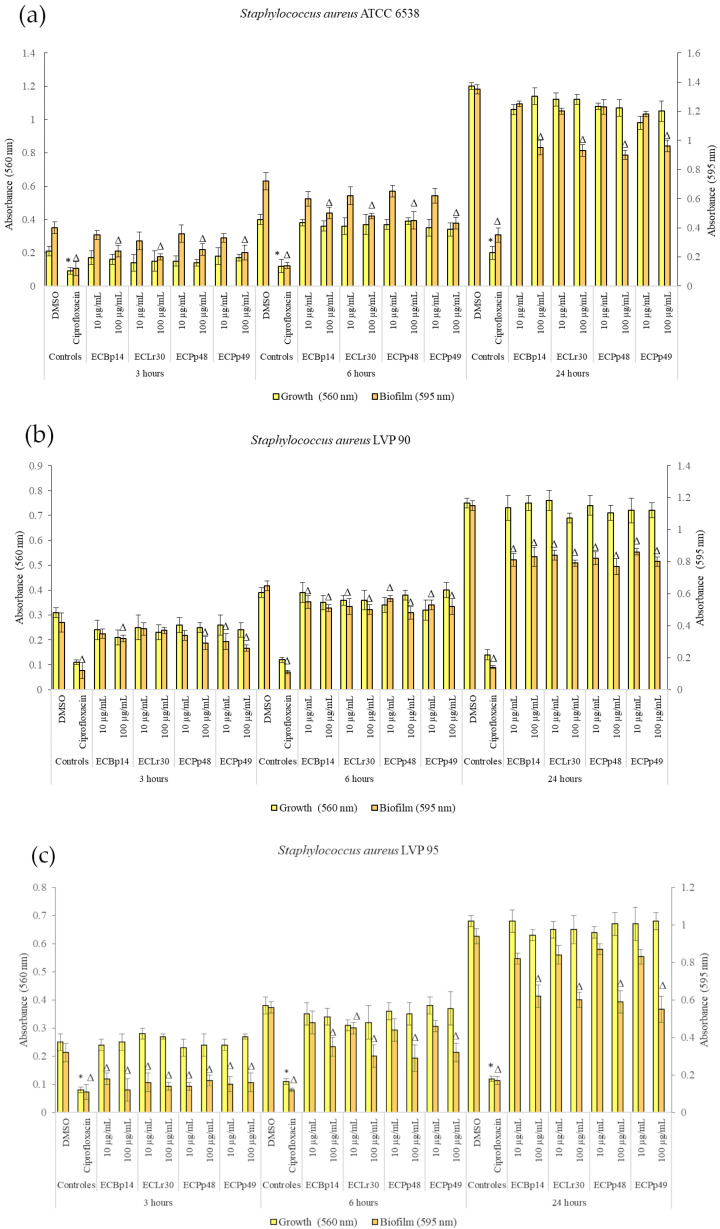
Biofilm formation by *Staphylococcus aureus* ATCC 6538 (**a**), LVP 90 (**b**), and LVP 95 (**c**) after 3, 6, and 24 h of incubation in the presence (10 and 100 μg/mL) and absence of the chloroformic extracts CEBp14, CELr30, CEPp48, CEPp49, and ciprofloxacin (5 μg/mL). Values represent the means ± SD of at least three independent experiments. ^Δ,^* values are significantly different with *p* ≤ 0.05 compared to the respective DMSO control.

Figure 1 shows the effect of the extracts on the growth and biofilm formation of the *Staphylococcus aureus* strains.

No significant growth inhibition was observed at 3 h, 6 h, or 24 h in the strains tested.

Regarding the biofilm of *S. aureus* ATCC 14028, the most significant inhibition was observed in the biofilm adhesion stage (3 h) with a dose-dependent effect, with a more significant inhibition being observed at a concentration of 100 µg/mL, with values of 40% (CEBp14), 50% (CELr30), 38% (CEPp48), and 49% (CEPp49). The biofilm inhibitory effect was lower after 6 h of incubation, with the most active extracts being CEPp48 and CEPp49 with 40% inhibition. In the case of the 24-h-old biofilm, all the extracts presented an inhibitory capacity of around 30% at a concentration of 100 µg/mL (Figure 1a).

Regarding the biofilm of *S. aureus* LVP 90, the inhibition remained relatively constant at 3, 6, and 24 h, with a dose-dependent effect but not an extract-dependent effect since the inhibition percentages were similar for the four extracts. This range was from 17% (10 µg/mL) to 38% (100 µg/mL) at 3 h, from 15% (10 µg/mL) to 26% (100 µg/mL) at 6 h, and finally from 25% (10 µg/mL) to 33% (100 µg/mL) at 24 h.

Figure 1c shows the effect of the four extracts on the growth and biofilm inhibition of the strain LVP 95. In the case of biofilms, the greatest inhibitory effect was observed in the biofilm adhesion stage (3 h) with a percentage of 44% and 63% for CEBp14, 50% and 56% for CELr30, from 47% and 56% for CEPp48, and 50% for CEPp49 at concentrations of 10 and 100 µg/mL, respectively. At 6 h, all extracts inhibited the biofilms by approximately 15% (10 µg/mL) to 48% (100 µg/mL). At 24 h, this effect remained constant and ranged from 10% to 40% for all extracts in the same concentration range.

### 2.2. Biofilm Disruption and Metabolic Activity of Cells within the Biofilm

**Figure 2:**
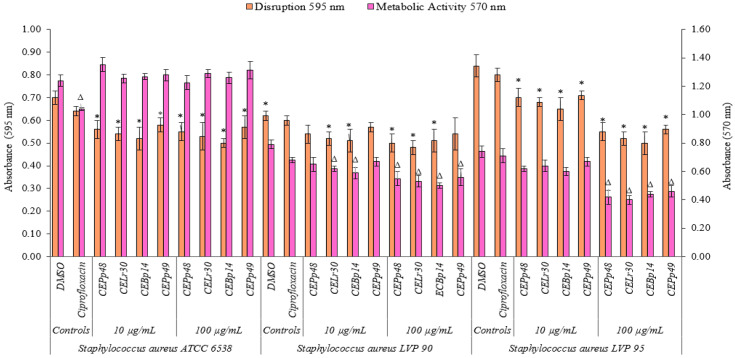
Biofilm disruption and biofilm metabolic activity of *Staphylococcus aureus* ATCC 6538, *Staphylococcus aureus* LVP 90, and *Staphylococcus aureus* LVP 95 after 24 h of incubation in the absence and presence (10 and 100 μg/mL) of chloroformic extracts (CEBp14, CELr30, CEPp48, and CEPp49) and ciprofloxacin (5 μg/mL). ^Δ,^* values are significantly different with *p* ≤ 0.05 compared to the respective DMSO control.

Figure 2 shows the effect of the chloroform extracts on the metabolic activity and their ability to eradicate a previously formed biofilm of *S. aureus* ATCC 6538, LVP90, and LVP95.

The CEBp14, CELr30, CEPp48, and CEPp49 extracts showed a disruption capacity between 17% and 29% for previously formed biofilms of *S. aureus* ATCC 6538. On the contrary, none of the extracts were able to inhibit the metabolic activity of the cells within the biofilms.

In the case of *S. aureus* LVP90, the extracts showed a higher capacity to inhibit metabolic activity concerning biofilm disruption. The four extracts inhibited the metabolic activity in a range of 15% to 37% for the concentration range of 10 to 100 µg/mL. There were only slight disruptions to the biofilms, ranging from 8% to 23% in the same concentration range.

For *S. aureus* LVP95, the percentages for both inhibition of metabolic activity and biofilm disruption presented similar values. For the inhibition of metabolic activity, these percentages were 14% (10 µg/mL) and 46% (100 µg/mL), and for disruption, these values were 15% (10 µg/mL) and 40% (100 µg/mL).

As can be seen, the effect of the extracts on the metabolic activity and the integrity of the *S. aureus* biofilms was strain-dependent.

### 2.3. Effect of the Extracts on the Hemolysin Activity of S. aureus

**Figure 3:**
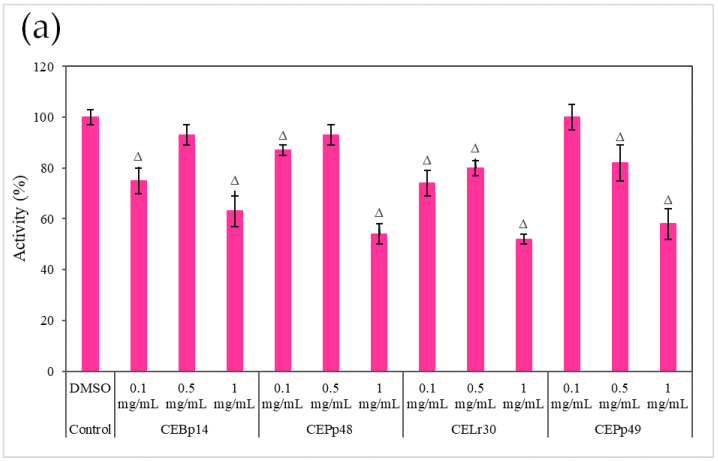

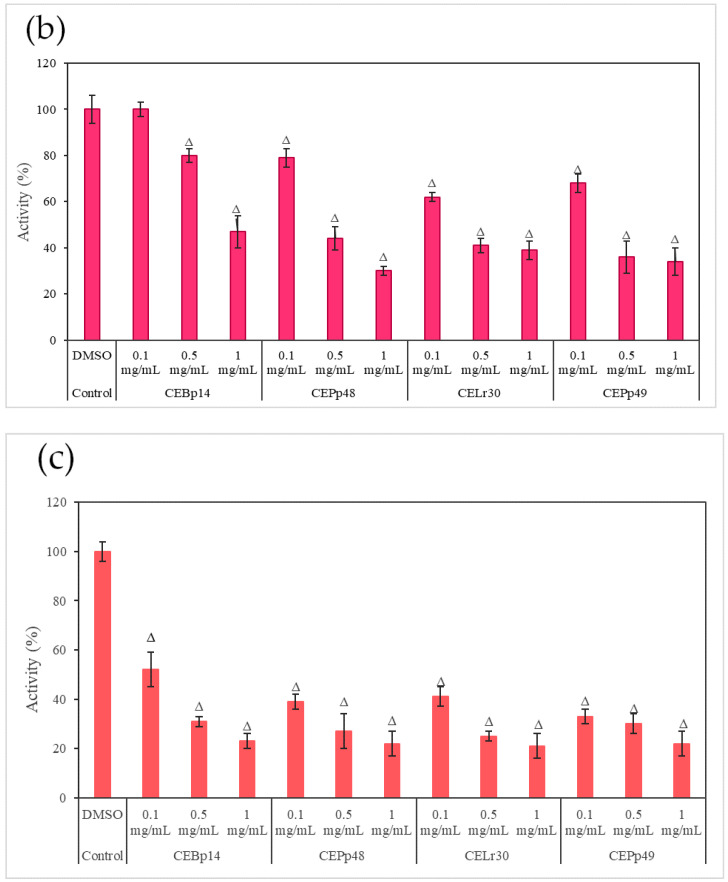
Hemolysin activity of (**a**) *S. aureus* ATCC 6538, (**b**) *S. aureus* LVP 90, and (**c**) *S. aureus* LVP 95 in the presence of 0.1 mg/mL, 0.5 mg/mL, and 1 mg/mL of CEBp14, CELr30, CEPp48, CEPp49 extracts. Δ indicates the samples that have significantly different hemolysin activities (*p* < 0.05) compared to the control with DMSO. The statistical analysis was performed by applying the Tukey test.

Figure 3 shows the effect of the evaluated extracts on the α-hemolysin activity of *S. aureus* ATCC 6538, LVP 90, and LVP 95.

A dose-dependent inhibition was observed in the case of *S. aureus* ATCC 6538 (Figure 3a). The extracts corresponding to *P. pentosaceous* LVP 48 (CEPp48) and *P. pentosaceous* LVP 49 (CEP49) presented a similar behavior, with an inhibition percentage of 60% at a concentration of 1000 µg/mL.

A dose-dependent but not extract-dependent inhibition was also observed in strain LVP 90 (Figure 3b). In the case of the CELr30, CEPp48, and CEPp49 extracts, no significant differences were observed in the inhibition of α-hemolysin activity by increasing the concentration of the extract from 500 µg/mL to 1000 µg/mL, unlike the CEBp14 extract where the difference was significant, going from inhibition values of 20% (at 500 µg/mL) to 53% (at 1000 µg/mL).

In LVP 95 (Figure 3c), a strong inhibition of hemolysin activity was observed from the lowest concentration tested. The inhibition values were from 50% (100 µg/mL) to 80% (1000 µg/mL) in the presence of all the extracts.

As can be seen from the graphs, the inhibitory effect of the extracts was strain-dependent, being higher in the *S. aureus* LVP 95 strain (isolated from milk) and lower in *S. aureus* ATCC 6538.

### 2.4. Effects of Extract Supernatants on Coagulase Activity of S. aureus Strains

In the *S. aureus* strain ATCC 6538, it was observed that the process of plasma clot formation started after 30 min of incubation in the control tube where plasma was inoculated with the culture with 1% DMSO (extract solvent). At 30 and 60 min, all the extracts in the range of concentrations tested (from 100 to 1000 µg/mL) prevented the formation of the plasma clot.

After 90 min, the clot formed in the control group was more extensive and consistent. At this time, all the extracts, except for ECBp14 at 100 µg/mL, showed the ability to delay clot formation, with only a small clot with little integrity. At 120 min, all the extracts at concentrations of 500 and 1000 µg/mL formed a coagulum that was less robust than that observed for the control. In summary, all the extracts showed the capacity to delay the formation of plasma clots in an effect that was not extract-dependent but dose-dependent after 90 and 120 min of incubation.

For the clinical isolate, *S. aureus* LVP 90, the plasma clot began to form after 60 min of incubation. All the extracts could delay the formation of the plasma clot in very similar behavior and with dose-dependent effects.

Finally, the LVP 95 strain showed the most marked inhibition effect on the formation of plasma clots of the three strains of *S. aureus* under study. After 120 min of incubation, the clot formed with the extracts at concentrations of 500 and 1000 µg/mL was small and fragmented, compared to the larger and more consistent clot of the DMSO control, indicating a decrease in coagulase activity.

### 2.5. Gas Chromatography–Mass Spectrometry Analysis

Eight known cyclic dipeptides and their isomers were characterized from the cell-free culture supernatants of CEBp14, CELr30, CEPp48, and CEPp49 by gas chromatography coupled with EI mass spectrometry (GC/EI-MS) (Table 1).

**Figure 4:**
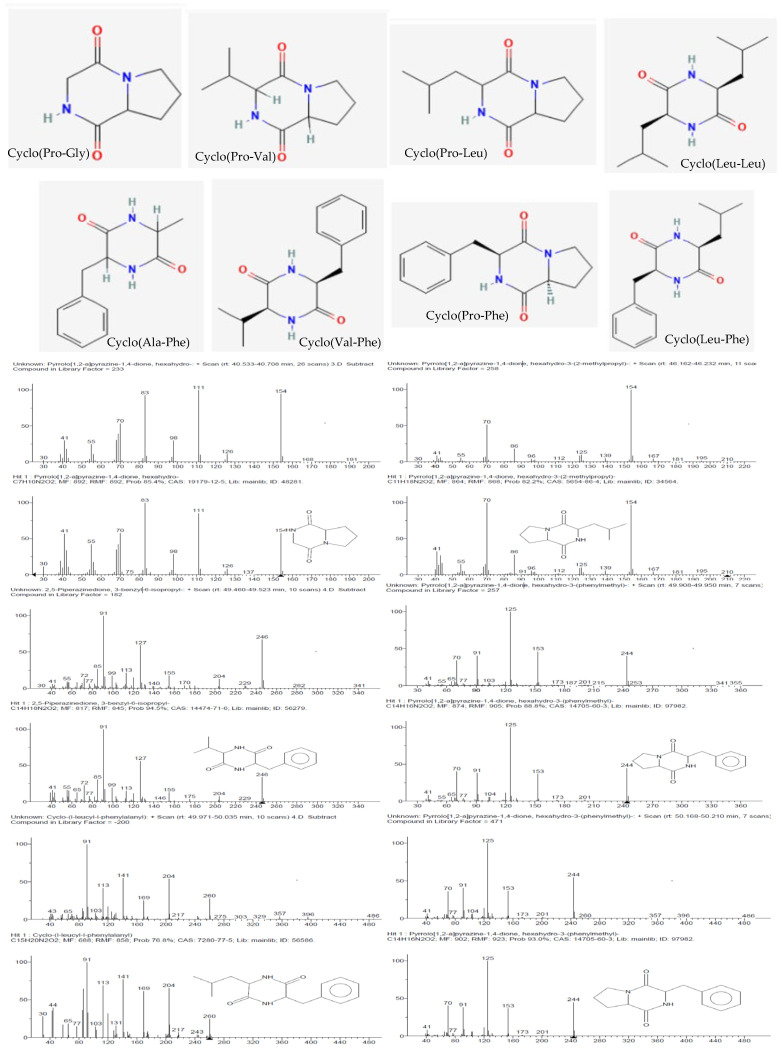
Structures (obtained from PubChem) and mass spectra of the 2,5-diketopiperazines (identified by comparison with the mass spectra in the NIST 11 library) present in the CEBp14, CELr30, CEPp48, and CEPp49 extracts.

The detected compounds showed a mass spectrum that was consisted with the mass spectra reported for 2,5-diketopiperazines (2,5-DKPs) (Figure 4), with the characteristic ions for the individual DKPs containing proline (m/z 70), valine (m/z 72), leucine/isoleucine (m/z 86), or phenylalanine (m/z 120) [14]. 2,5-DKPs derived from non-polar amino acids were found in the extracts since they were extracted using chloroform, a non-polar solvent. Polar DKPs could not be isolated using chloroform. The qualitative analysis showed that cyclo(Pro–Gly), cyclo(Pro-Leu), and cyclo(Pro-Phe) were detected in all samples (Table 1). From a quantitative point of view, the analysis of the chloroform extracts showed that the aromatic DKP cyclo(Pro-Phe) was the principal constituent (Table 1), followed by cyclo-proline with valine or leucine depending on the analyzed extract. The retention time and fragmentation pattern were consistent with the data found in the literature [15,16].

Although the absolute configuration was not determined, different enantiomers were found in the different bacterial cultures, which was attributed to the natural use of L- and D-amino acids by microorganisms or epimerization. It is interesting to note that the epimerization preferentially occurred at positions 6 and 9, and in previous works, it was observed that standard cyclo(L-Pro–L-Leu) epimerization was predominant (21%), followed by that of cyclo(L-Pro–D-Leu) (18%), cyclo(D-Pro–L-Leu), and cyclo(D-Pro–D-Leu) (both 11%) [17]. Specifically, cyclo(Pro-Leu) and cyclo(Pro-Phe) were identified in all samples at two different time points.

More than 73% of the compounds detected in the ECPp49 extract were 2,5-DKPs. Among these compounds, in addition to proline derivatives, phenylalanine derivatives were identified with alanine (1.39%) as well as valine (2.40%) and leucine (6.46%), which were detected in this analyzed samples.

Finally, other compounds were detected in all the bacteria cell-free supernatant extracts, such as 2-pyrrolidinone (0.2–0.5%) and hexadecanoic acid (0.4–1.1%).

### 2.6. Molecular Docking Calculations

#### 2.6.1. SarA

SarA functions as a global regulator, promoting the expression of the *agr* system. In a previous study [18], it was discovered that a series of four 2,5-DKP derivatives bind to SarA at two major binding sites: the DNA-binding motifs involving the Helix–Turn–Helix (HTH) region and the β-hairpin (winged) region, and the divalent cation-binding pocket. Not surprisingly, as the compounds detected in this work are also 2,5-DKP derivatives, the docking calculations found that these compounds bind to the binding sites mentioned above (Table 2).

#### Binding Sites among the DNA-Binding Motifs

**Figure 5:**
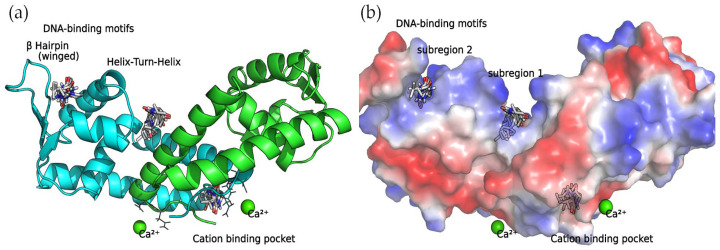
SarA homodimer binding sites. Bound compounds are shown as sticks. (**a**) The secondary structure of the protein is displayed as a cartoon where chain A is colored in cyan and chain B is colored in green. (**b**) Surface electrostatic potential calculated by PyMol. The positive charge is shown in blue, and the negative charge is shown in red.

The concave side of the SarA homodimer, characterized by a series of basic residues that form a positively charged, smooth surface (Figure 5), is likely to be the DNA-binding region [19]. Within this site, the docking calculations indicated that the compounds can bind two binding subregions: a wide and open pocket between the HTH of chain A and the interface of chain B (DNA subregion 1) and a narrow cavity between the winged and HTH motifs of chain A (DNA subregion 2).

**Figure 6:**
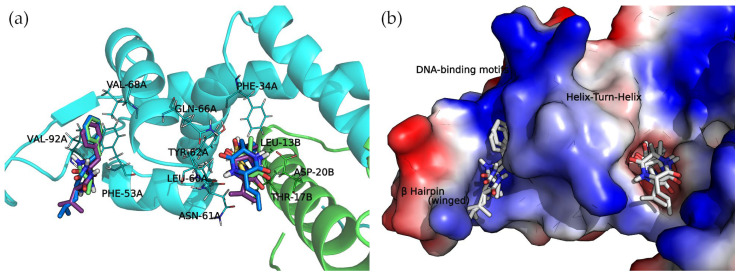
Docking complexes of 2,5-diketopiperazine derivatives in both DNA-binding site subregions. Bound compounds are displayed as sticks. (**a**) Molecular representation of the secondary structure elements (shown as cartoons) and side chains of interacting residues (shown as lines). Chain A is colored in cyan and chain B is in green. (**b**) Surface electrostatic potential calculated by PyMol. The positive charge is shown in blue, and the negative charge is shown in red.

The docked cyclo(Pro-Val) molecule located within subregion 1 of the SarA DNA-binding region is mainly stabilized by two hydrogen bonds formed by the side chain amide moiety of GLN-66A and the side chain of THR-17B (Figure 6).

Cyclo(Ala-Phe), cyclo(Val-Phe), and cyclo(Leu-Phe) extend the phenylalanine side chain towards hydrophobic residues such as PHE-34A, LEU-13B, and LEU-60A. These residues contribute to the interaction of protein chains in subregion 1, facilitating the formation of a dimer. Moreover, two hydrogen bonds strengthen this interaction: the side chain amide moiety of GLN-66A and backbone carbonyl oxygen of ASN-61A. Cyclo(Val-Phe) and cyclo(Leu-Phe) form additional hydrogen bonds with a side chain of residue ASP-20B that intensifies the binding. In addition, π-π interactions between PHE-34A and TYR-62A enhance the overall affinity. In subregion 2, the docking poses of these DKP derivatives are almost identical, with a high degree of molecular superposition, especially of the phenylalanine side chains, which can fit into the reduced volume of this pocket.

The interactions between 2,5-DKP and the amino acids of subregion 1 and 2 of the DNA-binding motifs are represented in Figures S1 and S2 of the Supplementary Materials.

#### Divalent Cation-Binding Pocket

**Figure 7:**
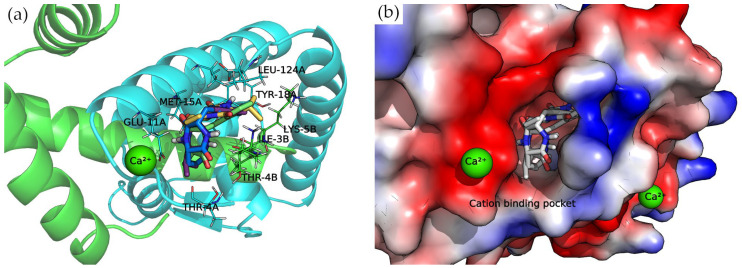
Docking complexes within cation-binding pocket. Bounded compounds are displayed as sticks. (**a**) Molecular representation of the secondary structure elements (shown as cartoon) and side chains of interacting residues (shown as lines). Chain A is colored in cyan and chain B in green. (**b**) Surface electrostatic potential calculated by PyMol. The positive charge is shown in blue, and the negative charge is shown in red.

The N-terminus of chain B and C-terminus of chain A, located at the convex side of the SarA dimer, present a negatively charged, smooth patch, as can be seen in the electrostatic potential surface (Figure 7) where a divalent cation is coordinated [19]. Next to this region is a pocket where the 2,5-DKP derivatives bind, according to our calculations (Figure 7).

All compounds presented a docking pose where the molecules are oriented deeper into the pocket, especially the three derivatives bearing a phenylalanine side chain. The latter compounds point this chemical moiety towards the TYR-18 amino acid of chain A, with which it can establish a T-shaped π-π interaction (Figure 7).

Moreover, the multiple van der Waals contacts and hydrogen bonds from the backbone atoms of the surrounding amino acids (ILE-3B, MET-15A, THR-4A, and LEU-124A) stabilize the docking complexes. All the interactions of each docking complex are represented as 2D diagrams in Figure S3 of the Supplementary Materials.

#### 2.6.2. AgrA

**Figure 8:**
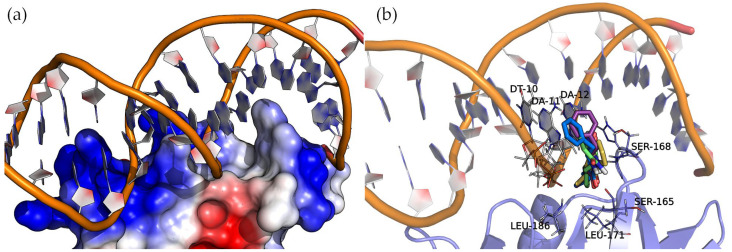
Molecular representation of AgrA (**a**) electrostatic potential surface and (**b**) binding sites detected by docking. Protein and DNA are displayed as cartoons, interacting residues as lines, and compounds as sticks. The positive charge is shown in blue, and the negative charge is shown in red.

AgrA forms part of a two-component transduction system. Upon activation, it functions as a transcription factor, controlling the expression of numerous virulence factors. Figure 8 shows the surface electrostatic potential of AgrA (PDB ID: 3BS1), where the positively charged areas (blue) bind with a short double-stranded DNA sequence. A closer inspection of the nucleic acid major groove allows us to see a protein protrusion that interacts with nitrogenous bases, leaving an empty pocket where compounds can bind. The docking of cyclic dipeptides revealed this pocket as a common binding site for all the studied compounds (Figure 8).

The four 2,5-DKP derivatives presented the same overall orientation, maximizing the interactions between the protein and the nucleic acids. The hydrogen bond interactions are listed in Table 3 and Figure S4 of the Supplementary Materials. The analysis of the binding energies (Table 3) revealed the same trend observed in the binding of these compounds to SarA, i.e., the larger the molecule, the higher the affinity. In addition to the hydrogen bond interactions, the hydrophobic side chains of the compounds (Val, Leu, and Phe) face towards the base of the protein pocket where the non-polar side chains of LEU-171 and LEU-186 provide a suitable hydrophobic environment to enhance the binding. Interestingly, the compounds with a phenylalanine common side chain extend the chemical moiety-facing nucleotide bases except for Cyclo(Ala-Phe), which is oriented with the phenylalanine side chain pointing to the bottom of the pocket. This pose results from stronger interactions that a larger side chain (phenylalanine > alanine) can establish in this binding pocket region.

## 3. Discussion

The growing number of bacteria resistant to antibiotics, causing infections that are difficult to eradicate, has led to the search for alternatives to combat these infections through mechanisms that do not generate selective pressures on bacteria, as compounds with bactericidal activity do.

Among these new approaches, one seeks to inhibit multiple virulence factors through the interruption of bacterial communication since it is a mechanism that does not result in the death of the bacteria, and therefore, would not produce selective pressures. This approach is called *quorum quenching*.

Various studies have been conducted with molecules with QS inhibitory activity from marine algae, fungi, and various bacterial genera. Among the latter, lactic acid bacteria have been studied for their ability to inhibit the QS of pathogenic bacteria such as *C. perfringens*, *C. difficile*, *E. coli*, *P. aeruginosa*, *B. cereus*, and *S. aureus*. Among the compounds that can repress the production of toxins, deregulate virulence genes, and inhibit biofilm formation are bacteriocins [20], biosurfactants [21], various enzymes [22], and organic acids [23]. However, multiple studies have used cell-free supernatants or crude extracts, but the components responsible for these properties have not been identified [24,25].

*S. aureus* is an opportunistic pathogen that causes food contamination and various diseases that can compromise human and animal life. Multiple *quorum* signaling pathways regulate the virulence of this bacteria, the most studied of which is the *agr* system. The autoinducing molecules of this system have a cyclic peptide structure. For this reason, finding antagonists that compete with them, thus blocking the activation of the bacterial QS, has become an attractive approach.

To our knowledge, few studies have focused on the search for compounds isolated from lactic acid bacteria, and none have focused on using bifidobacteria to inhibit QS and the virulence of *S. aureus* [26,27,28,29].

To combat the pathogenicity of these bacteria, four extracts obtained by chloroform extractions from the supernatant of cultures of three *Lacticaseibacillus* strains (*Ls. rhamnosus* LVP30, *P. pentosaceous* LVP48, and *P. pentosaceus* LVP49) and one belonging to the genus *Bifidobacterium* (*B. pseudolongum* LVP14) previously isolated from goat feces were used.

We evaluated the ability of these four extracts to inhibit biofilm formation at different growth stages, inhibit the metabolic activity of cells in a mature biofilm, and disrupt biofilms. Furthermore, the effect of the extracts on virulence factors produced and regulated by *S. aureus* QS, such as coagulase and the α-hemolysin toxin, was evaluated.

From these studies, it was observed that none of the extracts showed the ability to inhibit the bacterial growth of the *S. aureus* strains under study. However, they showed the ability to inhibit the formation of biofilms of the three strains under study, and this effect was dependent on the extract concentration used and not on the type of lactic acid bacteria. This effect is desirable since the absence of compounds with a bactericidal effect would prevent the generation of bacterial resistance due to the selective pressure exerted.

Other studies used various lactic bacteria (*Lactiplantibacillus plantarum*, *Lacticaseibacillus rhamnosus*, *Lactobacillus pentosus*, *Limosilactobacillus fermentum*, and *Lactobacillus gasseri*) isolated from goat milk to evaluate their antibacterial and antibiofilm capabilities against *P. aeruginosa* and *S. aureus* [30]. However, in these studies, this effect was due to the production of organic acids such as lactic acid, which reduces the pH of the medium, thus hindering the growth of pathogenic bacteria.

In this same study, the ability of the cell-free supernatant to disrupt a mature biofilm was determined, observing that the most significant disruption occurred in the 12 h biofilm. Also, the cell-free supernatant showed the ability to inhibit the metabolic activity of mature biofilms of *S. aureus* and *P. aeruginosa*. However, these properties decreased or disappeared when the cell-free supernatant was neutralized or thermally treated [30]. Similarly, multiple studies using cell-free supernatants of lactic acid bacteria of different origins demonstrated antibiofilm effects through the secretion of antagonistic substances such as surfactants, bacteriocins [20], organic acids [31], exopolysaccharides [32], enzymes [22], and hydrogen peroxide [33,34]. However, in our study, we took precautions to prevent the extraction of high-polarity molecules, such as organic acids, by employing a medium-polarity solvent. Therefore, the observed activities cannot be attributed to their presence. Nonetheless, chloroform extraction also eliminates polar DKPs.

The chemical identification of the molecules present in the chloroform extracts (CEBp14, CELr30, CEPp48, and CEPp49) using gas chromatography coupled with mass spectrometry revealed that the main compounds present in the four extracts were 2,5-DKPs (Cyclo-Pro-Gly, Cyclo-Pro-Val, Cyclo-Pro-Leu, Cyclo-Ala-Phe, Cyclo-Leu-Leu, Cyclo-Val-Phe, Cyclo-Pro-Phe, and Cyclo-Leu-Phe). Cyclic dipeptides, also known as DKPs or dipeptide anhydrides, are the simplest and most natural cyclic forms of peptides, commonly biosynthesized by a wide variety of organisms.

Our focus was on extracting small lipophilic molecules such as cyclic peptides. These peptides are highly favored for drug development owing to their low molecular weight (less than 500) and lipophilicity (LogP less than 5), both of which conform to established standards. Additionally, they typically possess fewer than 5 hydrogen-bond donors and 10 hydrogen-bond acceptors, further enhancing their suitability for pharmaceutical applications [35].

These compounds have already been found in the supernatant of lactic bacteria used in food fermentation and isolates from healthy infants’ feces. The structural similarity of 2,5-DKPs to peptides and their resistance to proteolysis, their privileged structure, and the fact that they follow the rule of analogues and can bind to a great diversity of receptors, they have become attractive scaffolds for the discovery of new drugs [36,37].

Our results on biofilm inhibition and disruption are in agreement with others in which it was reported that cyclic dipeptides at sub-MIC concentrations could inhibit the formation of biofilms of MRSA in the initial stages, reducing the formation of microcolonies and facilitating dispersion in mature biofilms that showed recalcitrance [38].

In the case of α-hemolysin, the four extracts (whose main components are 2,5-DKPs) were able to inhibit the production of this toxin in the three strains tested in a strain-dependent manner. Various studies have focused on the effects of various metabolites of lactic bacteria on α-toxin, such as those carried out by Jiang and collaborators, where *Lactobacillus helveticus* produced biosurfactants that could inhibit the hemolytic activity of *S. aureus* CMCC26003, in addition to anti-adhesive effects on its biofilms [27].

Coagulase, also known as agglutination factor, is what is known as microbial surface component recognition adhesive matrix molecules (MSCRAMMs) and is necessary for *S. aureus* to infect host tissues by initially promoting adhesion and subsequently the formation of a biofilm [39]. This study showed that extracts could delay the formation of plasma clots compared to the control group. We also observed a greater inhibition of biofilm formation by the extracts in our assays.

Within Staphylococci, the accessory gene regulator (*agr*) QS pathway stands out as a specialized route leading to the accumulation of signaling molecules and the activation of virulence factors such as cell wall-associated proteins and exotoxins. The *agr* activity has been associated with biofilm modulation. In addition, heightened *agr* expression corresponds to the enhanced production of several virulence factors [11]. Inhibiting AgrA represents a pragmatic strategy to attenuate virulence, rendering the pathogen more susceptible to the host immune response and antibiotic treatments [40]. The quorum regulator SarA found in *S. aureus* is known to enhance the expression of numerous virulence factors, including those involved in biofilm formation. This process plays a crucial role in the pathogenesis of the bacteria and its ability to evade the host immune system during later stages of growth. Consequently, interfering with the activity of the SarA protein could potentially lead to a reduction in biofilm formation and the expression of virulence factors [41].

2,5-DKPs have been found in the methanolic extract of *Limosilactobacillus reuteri* RC-14, which were identified as cyclo(Tyr-Pro) and cyclo(Phe-Pro) (also present in our extracts) and found to have the ability to inhibit the expression of the *agr* system of *S. aureus*, acting as competitive inhibitors of the autoinducing peptides that activate the P3 promoter of the system [42]. On the other hand, the expression of virulence genes, such as the gene (*hla*) that encodes alpha toxin and SarA, were significantly down-regulated when exposed to cyclic dipeptides [37].

It has also been proven that components such as lipoteichoic acid from *Lp. plantarum* KCTC10887BP can induce the production of autoinducer type 2 (AI-2), repressing genes involved in biofilm production in *S. aureus* [28].

Regarding the other compounds identified in the extracts, it has been reported that hexadecanoic acid, also known as palmitic acid, at a concentration of 100 µg/mL is unable to inhibit biofilm formation by *S. aureus* MSSA 6538 [43]. However, it appears to be effective against the growth of Gram-negative bacteria such as *Escherichia coli* and *Klebsiella pneumoniae* at concentrations greater than 500 µg/mL [44] and could inhibit biofilm formation by *Vibrio* spp. at 100 µg/mL [45]. On the other hand, to our knowledge, 2-pyrrolidinone was not tested for its ability to inhibit bacterial biofilm.

## 4. Materials and Methods

### 4.1. Bacterial Strains and Growth Conditions

The pathogenic strains used in this study included *S. aureus* ATCC 6538 obtained from American Type Culture Collection (Manassas, VA, USA) isolated from a human lesion, *S. aureus* LVP 95 isolated from contaminated milk by Bacteriology Department of the Faculty of Biochemistry, Chemistry, and Pharmacy at the National University of Tucumán (San Miguel de Tucumán, Argentina), and *S. aureus* LVP 90 isolated from a chronic skin lesion (Hospital Ángel Cruz Padilla, San Miguel de Tucumán, Argentina) that is resistant to methicillin (30 μg), gentamicin (30 μg), and azithromycin (5 μg). The bacteria were cultured in Müller–Hinton (MH) medium (Laboratorios Britania, Buenos Aires, Argentina) at 37 °C under aerobic conditions with agitation at 150 rpm.

The lactic acid bacteria strains used were *Lacticaseibacillus rhamnosus* LVP 30 (goat origin, GenBank SUB5337873 LVP 30 MK656097), *Pediococcus pentosaceous* LVP 48 (goat origin, GenBank SUB5513902 LVP 48 MK825574), *P. pentosaceous* LVP 49 (goat origin, GenBank SUB5514976 LVP 49 MK825576), which were cultured at 37 °C in LAPTg broth (peptone, 15 g/L; tryptone, 10 g/L; yeast extract, 10 g/L; glucose, 10 g/L; Tween 80, 0.1%, *v*/*v*) under microaerophilic conditions and *Bifidobacterium pseudolongum* LVP 14 (goat origin, GenBank SUB4998733 LVP 14 MK353737) was cultured in MRS medium (Britania, Argentina) with the addition of 0.05% cysteine under anaerophilic conditions generated by Mitsubishi Gas Chemical envelopes (Tokyo, Japan).

These strains were deposited in the culture collection of the Laboratory of Research of Added Value of Regional Products and Foods (LVP) at INBIOFAL (Instituto de Biotecnología Farmacéutica y Alimentaria, San Miguel de Tucumán, Argentina).

### 4.2. Preparation of Extracts from Lactic Bacterial Culture Supernatants

Each lactic acid bacterium was cultured in 5 L of the appropriate culture medium for 48 h at 37 °C under microaerophilic or anaerobic conditions. Following incubation, the supernatants were obtained by centrifugation at 3500 rpm (2193× *g*) for 15 min at 4 °C and subsequently filtered. The resulting supernatant filtrates underwent three extractions with an equal volume of chloroform. During each extraction, the phases were allowed to separate, and in the case of emulsion formation, phase separation was facilitated by the addition of NaCl (0.05 g/L). The chloroform phases were concentrated under reduced pressure using a vacuum rotary evaporator until dry. The resulting chloroform extracts, designated as CEBp14, CELr30, CEPp48, and CEPp49, were stored in refrigerated caramel-colored bottles at 4 °C until further use.

### 4.3. Bioassay of Antibacterial Activity

The inhibition of bacterial growth was determined using a previously described protocol [18]. Aliquots of 5 µL of each extract (at concentrations of 10 µg/mL and 100 µg/mL) were placed in each well and mixed with 195 µL of the *S. aureus* ATCC 6538, LVP 90, or LVP 95 bacteria (OD560nm = 0.08 ± 0.02). As a growth control, a bacterial culture with 5 µL of DMSO (2.5% final concentration in the well) instead of the extract was employed. The positive control was ciprofloxacin (5 µg/mL).

### 4.4. Antibiofilm Activity

#### 4.4.1. Biofilm Formation Assay

A previously described technique [18] was used to quantify biofilm formation. The biofilm formed by *S. aureus* ATCC 6538, LVP 90, and LVP 95 was determined after 3, 6, and 24 h of incubation in the presence of 10 µg/mL and 100 µg/mL of the CEBp14, CELr30, CEPp48, and CEPp49 extracts. Briefly, a bacterial suspension (OD560nm = 0.08 ± 0.02) was placed in the wells of a 96-well microplate and treated for 24 h with the extracts at concentrations of 10 and 100 µg/mL.

#### 4.4.2. Biofilm Disruption Assay

Aliquots of 200 µL of the culture (OD560nm 0.08 ± 0.02) of the tested strains were seeded into a 96-well microplate and incubated for 24 h to allow biofilms to form in the wells. The biofilm formed after that time was washed twice with phosphate-buffered saline (PBS) and air-dried. Then, 5 μL of the extracts and 195 μL of PBS were added to each well, achieving final concentrations of 10 and 100 µg/mL for each extract (CEBp14, CELr30, CEPp48, and CEPp49). The remaining biofilm in the wells after an additional 24 h of treatment was determined by crystal violet staining [18]. As controls, 5 µL of 2.5% DMSO or ciprofloxacin (5 µg/mL) was used.

#### 4.4.3. Biofilm Metabolic Activity Assay

The remaining metabolic activity of the bacteria from the previously formed biofilm treated with 10 µg/mL and 100 µg/mL of each extract (see Biofilm Disruption Assay) was determined using a previously described protocol [18]. In the final step, 100 µL of an MTT solution (500 µg/mL) was added. After 6 h, the formed formazan purple was dissolved with DMSO and measured spectrophotometrically at 570 nm.

### 4.5. Virulence Factor Inhibition

#### 4.5.1. Hemolysin Assay

Hemolysin activity was determined by red cell lyses, as described previously, with some modifications [16]. Briefly, all *S. aureus* strains were grown in MH broth for 24 h at 37 °C in the presence of different concentrations of the extracts (10, 100, and 500 µg/mL). After 24 h of incubation, 200 μL of the cultures (containing hemolysin) were added to Eppendorf tubes containing 25 μL of red blood cells and 775 μL of PBS buffer (pH = 7.4) and were incubated at 37 °C for 1 h with agitation. After this incubation period, the samples were centrifuged in an Eppendorf centrifuge at 3000 rpm for 10 min, and then the hemoglobin released from the lysis of red blood cells was quantified spectrophotometrically at 450 nm using a microplate reader. The positive control for 100% hemolysis was 1% Triton X-100, and the negative control was the cultures incubated with DMSO 1% (extract solvent).

#### 4.5.2. Coagulase Assay

The extracts’ capacity to delay the clot formation caused by the *S. aureus* ATCC 6538, LVP 90, and LVP 95 strains was determined following the a previously detailed protocol [18]. The CEBp14, CELr30, CEPp48, and CEPp49 extracts were used at concentrations of 10, 100, and 500 µg/mL. A control culture was carried out with 1% DMSO.

### 4.6. Gas Chromatography–Mass Spectrometry Analysis

GC–MS analysis was carried out using a 5977A Agilent mass spectrometer (Agilent Technologies, Santa Clara, CA, USA) and a gas chromatograph (Agilent 7890B, Agilent Technologies) apparatus equipped with an Agilent HP-5MSi (30 m long and 0.25 mm i.d. with 0.25 µm film thickness) capillary column (95% dimethylpolysiloxane/5% diphenyl). The column temperature program was 60 °C for 5 min, with 3 °C/min increases to 180 °C, then 20 °C/min increases to 280 °C, which was maintained for 10 min. The carrier gas was helium at a 1 mL/min flow rate. Split mode injection (ratio 1:30) was employed. Mass spectra were taken over the m/z range of 30–650 with an ionizing voltage of 70 eV. MS identified the resulting individual compounds, and their identity was confirmed by comparison of their mass spectra with data available in the NIST 11 mass spectral library and the literature.

### 4.7. Molecular Docking Calculations

Molecular docking studies of the 2,5-DKP compounds with the two proteins of *S. aureus*, SarA (PDB ID: 2FNP) [19] and ArgA (PDB ID: 3BS1) [46], were performed using Autodock 4.2 [47]. Protein structures were prepared by adding missing heavy atoms and hydrogen atoms. The PDB2PQR Server [48,49] was used to assign protonation states to titratable amino acids according to a pH of 7.4. Kollman charges were added, and non-polar hydrogen atoms were merged using the protein preparation routine of Autodock Tools. Similarly, the compounds (PubChem IDs 98951, 139767, 13783105, and 7076347) were prepared with the ligand preparation procedure of Autodock Tools (version 1.5.6) [48].

The docking setup involved two procedures: (a) blind docking to locate potential binding sites through the whole protein surface and (b) a regular docking calculation. The latter was accomplished by placing a 70 × 70 × 70 grid box with a spacing of 0.375 Å in the protein regions revealed by blind docking. A Lamarckian genetic algorithm (GA) requested twenty-five million energetic evaluations to provide 200 docking poses for each compound. Each conformation was clustered using a root mean square deviation value of 2 Å. The highest binding energy (more negative) and largest cluster population were used as criteria to select the best-docked conformation. Ligplot+ [50] 2D diagrams of the interactions between proteins and each compound. 3D visualization and analysis were performed with PyMol (version 1.8.4).

### 4.8. Statistical Analysis

The results are the average of at least three independent experiments ± standard deviation (SD). The data obtained were analyzed statistically with the INFOSTAT Analytical Software (Universidad Nacional de Córdoba, Córdoba, Argentina) for Windows (version 2015). Differences in the mean absorbance values of the samples were evaluated by analysis of variance (ANOVA). The Tukey test was used to compare pairs. *p* < 0.05 was considered statistically significant.

## 5. Conclusions

This study highlights the inhibitory effects of lactic acid bacterial supernatants on the formation of biofilms and virulence factors in *S. aureus*. These inhibitory effects occurred at concentrations that do not compromise the growth of the pathogen, avoiding the bacterial resistance that normally occurs with antibiotic compounds. The molecular docking analysis revealed an interaction of 2,5-DKPs, present at high concentrations in the cell-free extracts, with key proteins in *S. aureus* suggesting the importance of these compounds in controlling the virulence factors regulated by QS. Therefore, the study contributes to the objective of finding alternatives to antibiotics and promoting the use of products that naturally control the virulence of pathogens.

## Figures and Tables

**Table 1:** Diketopiperazines in chloroform extracts of cell-free lactic acid bacteria supernatants.

Rt (min)	DKPs	MW	ECBp14 Area (%)	ECLr30 Area (%)	ECPp48 Area (%)	ECPp49 Area (%)
40.60	Cyclo(Pro-Gly)	154	4.03	1.55	3.15	2.87
42.92	Cyclo(Pro-Val)	196	16.94	9.92	13.09	11.21
43.64	Cyclo(Pro-Val)	196		0.47	0.68	0.64
45.84	Cyclo(Pro-Leu)	210	8.70	6.34	8.60	6.07
46.27	Cyclo(Pro-Leu)	210	9.51	10.51	16.64	12.65
46.34	Cyclo(Leu-Leu)	226				7.56
48.66	Cyclo(Ala-Phe)	218	0.25			1.39
48.82	Cyclo(Leu-Leu)	226	4.21			
49.48	Cyclo(Val-Phe)	246				2.40
49.92	Cyclo(Pro-Phe)	244	1.05	1.18	2.05	2.08
49.99	Cyclo(Leu-Phe)	260				6.46
50.19	Cyclo(Pro-Phe)	244	18.58	26.72	27.98	20.59
Total			63.27	56.69	72.19	73.92

Rt = retention time (min); the absolute configuration of diketopiperazines was not determined. MW = molecular weight; Area (%) = relative peak areas of DKPs detected in the chloroform extracts.

**Table 2:** Selected binding sites according to binding free energy and cluster population for each 2,5 diketopiperazine docked onto SarA (PDB ID: 2FNP).

Compound	Union Free Energy (kcal/mol) (Calculated by Autodock 4.2)		
	DNA, Subregion 1	DNA, Subregion 2	Cation Pocket
Cyclo(Pro-Val)	−5.45	-	−5.72
Cyclo(Ala-Phe)	−5.98	−6.10	−6.32
Cyclo(Val-Phe)	−6.50	−6.89	−7.17
Cyclo(Leu-Phe)	−7.05	−7.24	−7.39
Cyclo(Pro-Gly)	−4.94	−4.9	−4.89
Cyclo(Leu-Pro)	−6.28	−5.2	−6.09
Cyclo(Leu-Leu)	−6.12	−5.91	−5.95
Cyclo(Phe-Pro)	−6.45	-	−6.98

**Table 3:** Docking energy and main interactions of each 2,5 diketopiperazine with AgrA (PDB ID: 3SB1).

Compound	Interaction	Distance (Å)	Autodock 4.2 Binding Free Energy (kcal/mol)
Cyclo(Pro-Val)	DA-12, H-don	2.0	−5.72
	DA-12, H-acc	2.3	
	SER-165, H-don	2.7	
	SER-168, H-don	2.2	
Cyclo(Ala-Phe)	DA-12, H-don	3.1	−6.32
	SER-165, H-acc	1.9	
	SER-168, H-don	2.2	
	HIS-169, H-don	2.0	
Cyclo(Val-Phe)	DA-12, H-acc.	2.6	−7.17
	DA-12, H-don.	2.4	
	SER-168, H-don.	2.1	
	SER-165, H-acc.	2.2	
	SER-165, H-don	2.4	
Cyclo(Leu-Phe)	DA-12, H-acc.	2.3	−7.39
	DA-12, H-don.	2.2	
	SER-168, H-don.	3.2	
	SER-165, H-acc.	2.1	
	SER-165, H-don	2.4	
Cyclo(Pro-Gly)	DA-12, H-don	2.8	−5.16
	SER-168, H-don	2.1	
	SER-165, H-don	2.2	
	SER-165, H-acc	2.2	
Cyclo(Leu-Pro)	DA-12, H-acc	2.3	−5.97
	DA-12, H-don	1.9	
	SER-168, H-don	2.4	
	SER-165, H-don	3.0	
Cyclo(Leu-Leu)	DA-12, H-acc	2.1	−6.93
	DA-12, H-don	2.2	
	SER-168, H-don	2.8	
	SER-165, H-acc	2.3	
Cyclo(Phe-Pro)	DA-11, H-acc	1.9	−6.80
	DA-12, H-don	2.2	
	SER-168, H-don	2.4	

## Data Availability

Data will be made available on request.

## Supplementary Materials

The following supporting information can be downloaded at: https://www.mdpi.com/article/10.3390/antibiotics13040297/s1. Figure S1: Interaction in the form of a 2D diagram of the best docked conformations of the 2,5-diketopiperazines (a) Cyclo(Pro-Val), (b) Cyclo(Ala-Phe), (c) Cyclo(Val-Phe), and (d) Cyclo(Leu-Phe) in the DNA-binding motif subregion 1 of the SarA protein. Hydrogen bonds are shown as green dashed lines and hydrophobic contacts as spline curves. Figure S2. Interaction in the form of a 2D diagram of the best docked conformations of the 2,5-diketopiperazines (a) Cyclo(Ala-Phe), (b) Cyclo(Val-Phe) and (c) Cyclo(Leu-Phe) in subregion 2 of the DNA-binding motifs of SarA protein. Hydrogen bonds are shown as green dashed lines and hydrophobic contacts as spline curves. Figure S3. Interaction in the form of a 2D diagram of the best docked conformations of the 2,5-diketopiperazines (a) Cyclo(Pro-Val), (b) Cyclo(Ala-Phe), (c) Cyclo(Val-Phe), and (d) Cyclo(Leu-Phe) in the cation pocket site of the SarA protein. Hydrogen bonds are shown as green dashed lines and hydrophobic contacts as spline curves. Figure S4. Docking poses of each 2,5-diketopiperazine in AgrA. (a) Cyclo(Pro-Val), (b) Cyclo(Ala-Phe), (c) Cyclo(Val-Phe) and (d) Cyclo(Leu-Phe). DNA is represented as cartoons, compounds as sticks, and interacting amino acids as lines. Hydrogen bonds appear as yellow dotted lines.
