# Supplementary material for: Restraining Staphylococcus aureus Virulence Factors and Quorum Sensing through Lactic Acid Bacteria Supernatant Extracts
Source: Antibiotics (Basel). 2024 Mar 25;13(4):297. doi: 10.3390/antibiotics13040297 (PMC11047364; doi:10.3390/antibiotics13040297)
Supplement: Supplementary file 1 [file antibiotics-13-00297-s001.zip › antibiotics-2921753-supplementary.pdf]

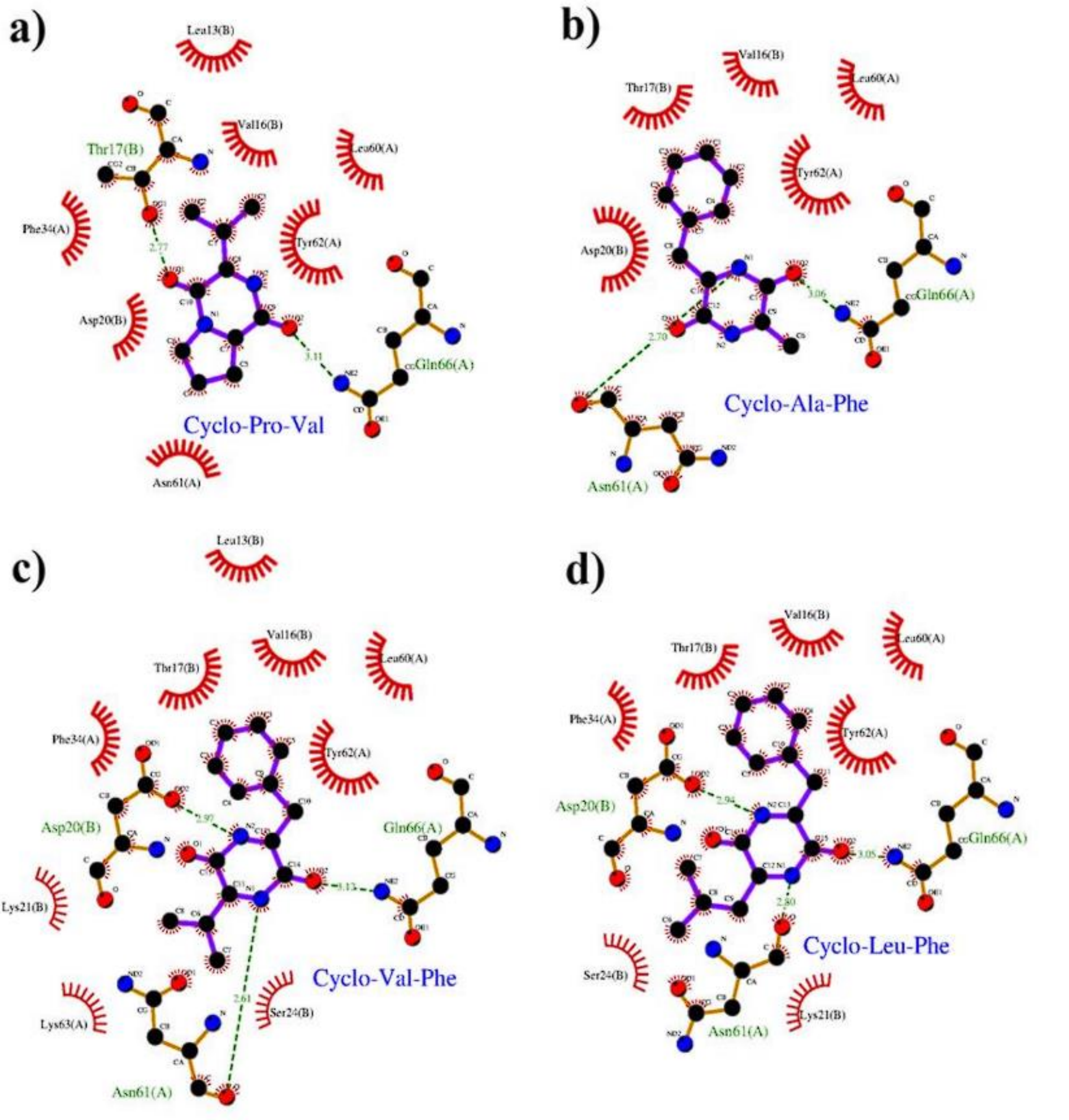

Figure S1. Interaction in the form of a 2D diagram of the best docked conformation of the 2,5-diketopiperazines: a) Cyclo(Pro-Val), b) Cyclo(Ala-Phe), c) Cyclo(Val-Phe), and d) Cyclo(Leu-Phe) in the DNA binding motif subregion 1 of the SarA protein. Hydrogen bonds are shown as green dashed lines and hydrophobic contacts as spline curves.

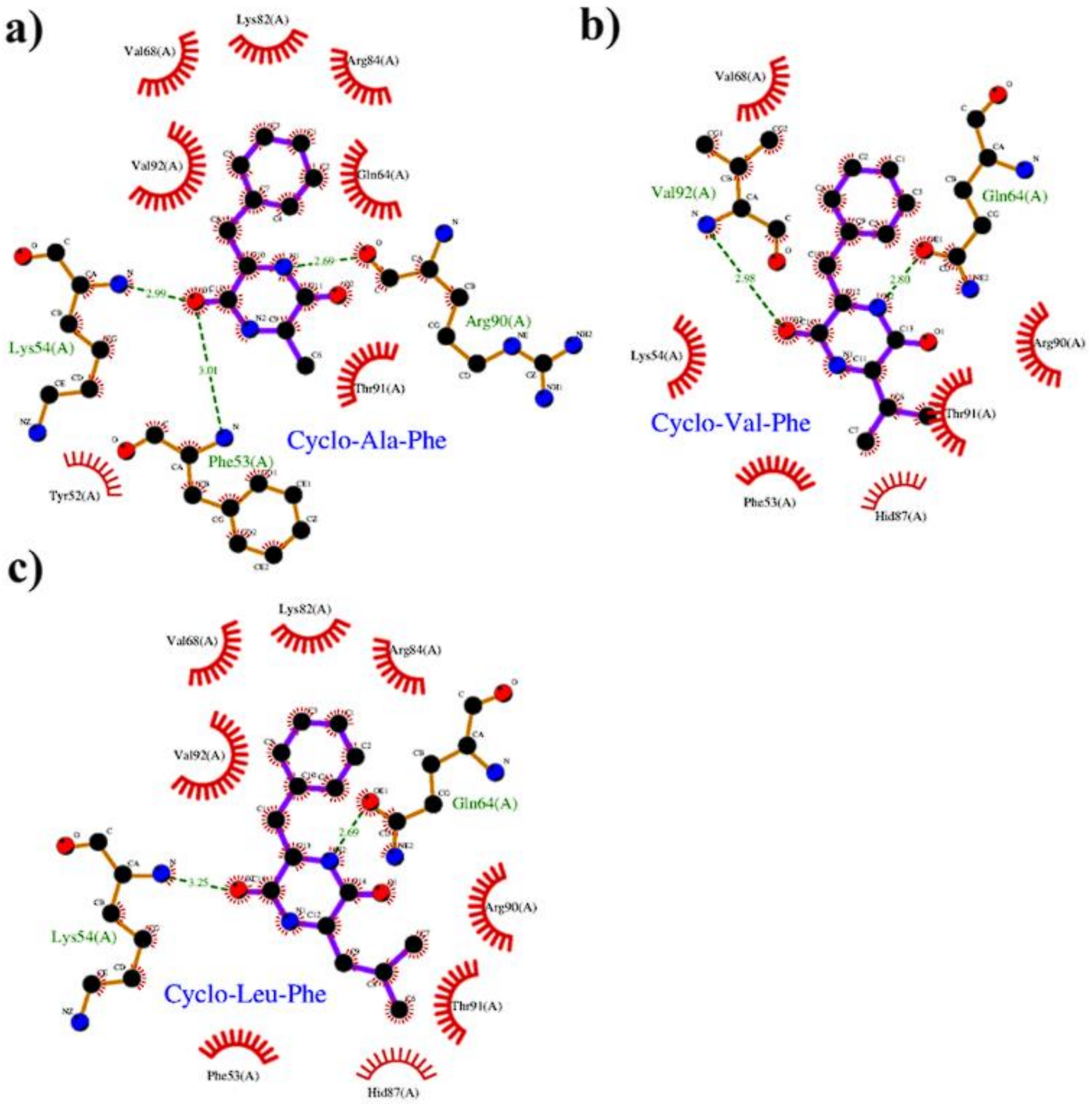

Figure S2. Interaction in the form of a 2D diagram of the best docked conformation of the 2,5-diketopiperazines: a) Cyclo(Ala-Phe), b) Cyclo(Val-Phe) and c) Cyclo(Leu-Phe) in subregion 2 of the DNA binding motifs of SarA protein. Hydrogen bonds are shown as lines green dashed and hydrophobic contacts as spline curves.

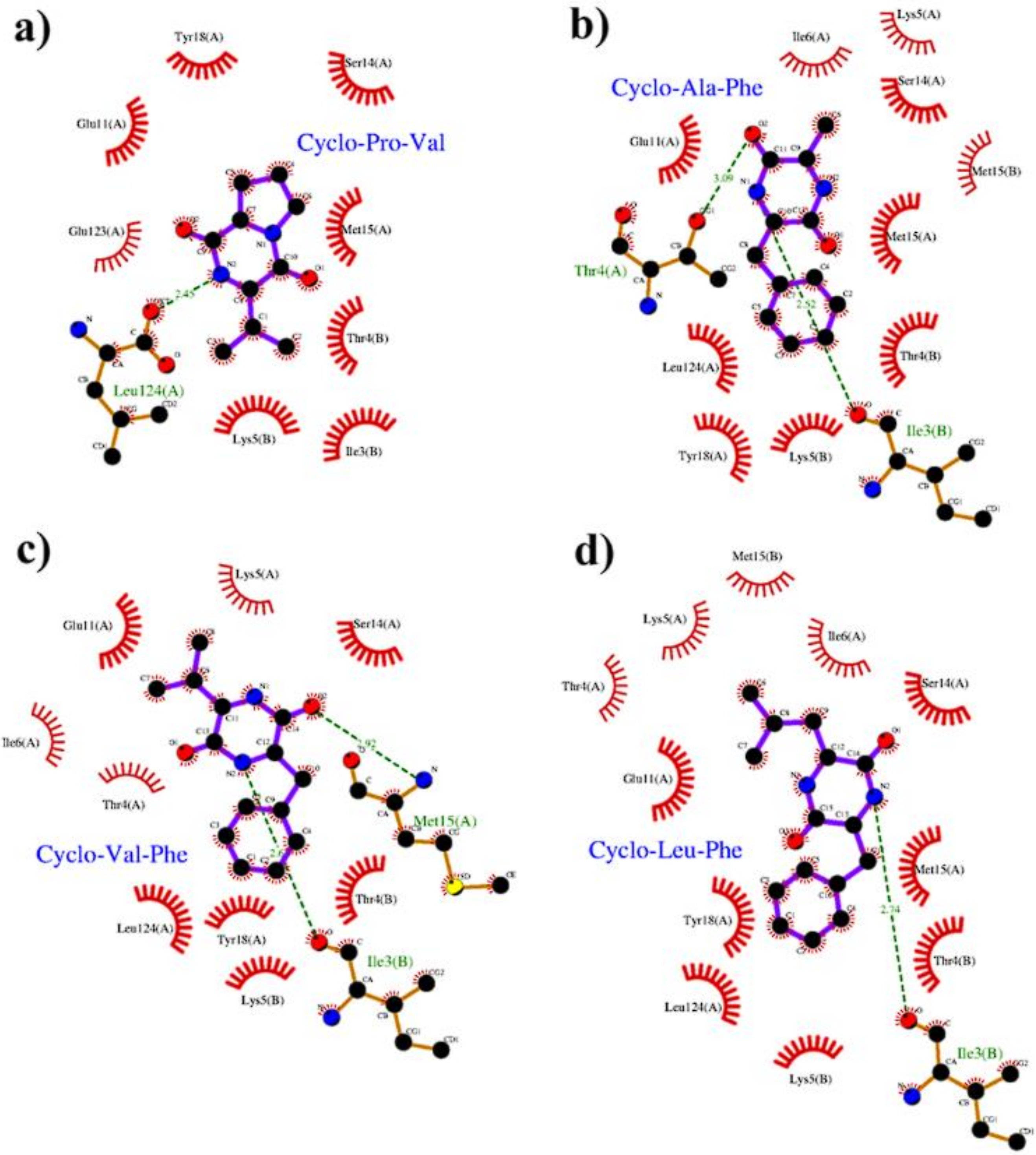

Figure S3. Interaction in the form of a 2D Diagram of the best docked conformation of the 2,5- diketopiperazines: a) Cyclo(Pro-Val), b) Cyclo(Ala-Phe), c) Cyclo(Val-Phe), and d) Cyclo(Leu-Phe) in the cation pocket site of the SarA protein. Hydrogen bonds are shown as lines green dashed and hydrophobic contacts as spline curves.

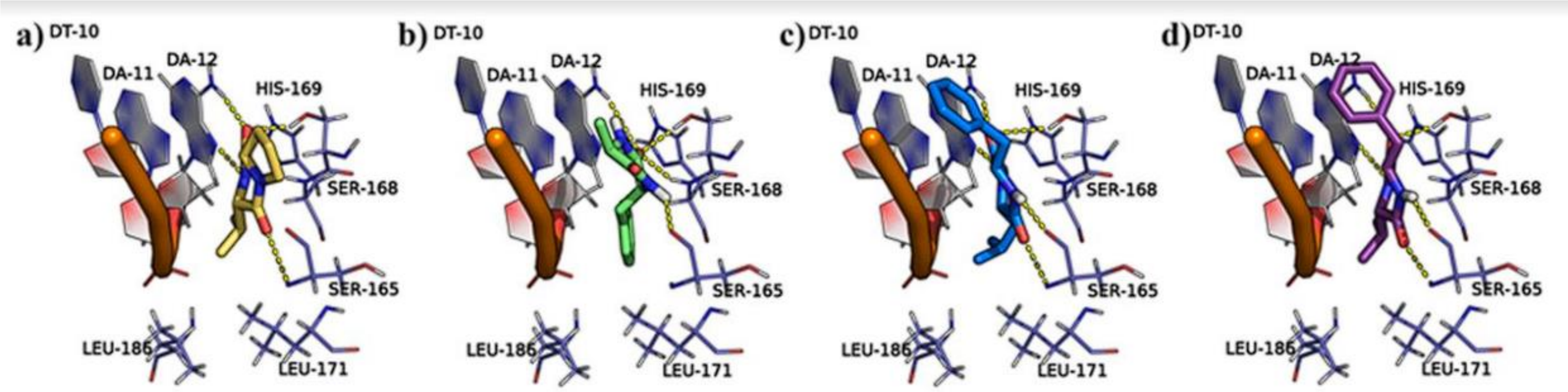

Figure S4. Docking poses of each 2,5-diketopiperazine in AgrA. a) Cycle(Pro-Val), b) Cycle(Ala-Phe), c) Cycle(Val-Phe) and d) Cycle(Leu- Phe). DNA is represented as cartoons, compounds as sticks, and interacting amino acids as lines. Hydrogen bonds appear as yellow dotted lines.
